# Fetal health state detection method based on parameters efficient ensembling of deep learning

**DOI:** 10.3389/fpubh.2026.1808284

**Published:** 2026-05-11

**Authors:** Weiwei Yin, Zhengyuan Shen, Zhenbo Cheng, Chun Feng, Guoquan Sun

**Affiliations:** 1 Hangzhou Red Cross Hospital, Hangzhou, Zhejiang, China; 2 College of Computer Science and Technology and College of Software, Zhejiang University of Technology, Hangzhou, Zhejiang, China; 3 The Second Affiliated Hospital, Zhejiang University School of Medicine, Hangzhou, Zhejiang, China

**Keywords:** cardiotocograph, clinical application, deep learning, ensemble learning, piecewise linear encoding

## Abstract

**Background:**

The classification of cardiotocography (CTG) can assist obstetricians in assessing the health status of the fetus. However, traditional fetal heart rate monitoring data has the problem of strong subjectivity in manual interpretation, and deep learning models have poor representation ability on tabular data.

**Methods:**

This study proposed PLE-TabM, a tabular deep learning model combined piecewise linear encoding (PLE) and efficient weight integration. We used the public CTG dataset as the research subject. The PLE was used to improve the perception ability of feature segment intervals and TabM integrates multiple Multi-Layer Perceptron (MLP) weak classifiers.

**Results:**

The experimental results of fetal health status classification demonstrated that the performance of the PLE-TabM algorithm exceeding traditional machine learning methods. Its accuracy reached 95.77% and macro averaged F1 score reached 93.83%. Meanwhile, Gradient SHapley Additive exPlanations (Gradient SHAP) was used to analyze the feature importance that affects the classification. Finally, the algorithm was verified on 50 clinical patients.

**Conclusions:**

This study combines efficient tabular learning with interpretability analysis and applies it to the CTG classification problem, providing a reliable and objective tool to assist obstetricians in fetal monitoring and clinical decision making.

## 1 Introduction

Perinatal care is the core focus of modern obstetrics. Fetal cardiotocograph monitoring is the primary non-invasive method for assessing the fetal health status in the uterus and identifying potential risks of fetal distress. It integrates indicators such as fetal heart rate variability, acceleration, deceleration, and uterine contractions. It can reflect the fetal oxygenation status and the function of the autonomic nervous system. It plays a crucial role in guiding the selection of the timing for cesarean section and during labor monitoring in clinical interventions (1). However, the traditional interpretation of CTG heavily relies on the experience of clinicians, resulting in significant differences among observers.

Especially in cases where the differentiation between normal, suspect, and pathological patterns is ambiguous. This subjectivity often leads to excessive intervention or missed diagnoses, both of which pose risks to the maternal and infant outcomes (2). Meanwhile, in different countries and at different times, the standard for diagnosing fetal distress in clinical settings vary. Kling et al. (3) compared the clinical efficacy of major CTG interpretation standards including FIGO (4), NICE (5), and SWE (6). The results showed that the NICE 2022 version standard demonstrated the highest diagnostic sensitivity in assessing the intrauterine condition of the fetus.

In the task of classifying tabular data, XGBoost often stands out as the top method in Kaggle's machine learning competitions (7). Chen et al. (8) proposed the WRF algorithm based on random forest, which can perform classification on the CTG dataset with imbalanced positive and negative samples. Feng et al. (9) integrated multiple basic machine learning methods and achieved an accuracy of 95.39%. Abiyev et al. (10) proposed adding fuzzy rules to neural networks for classification. Compared to traditional machine learning methods, multimodal large language models (LLM) also demonstrate their ability to interpret CTG signals and images. Psilopatis et al. (11) and Gumilar et al. (12) conducted recognition experiments on CTG images using multiple LLM agents. The results showed that directly inputting prompts into the LLM did not yield good results. Sun and Hu (13) preset the LB, AC, DP and other indicators for individual agents to identify them separately. Then he used aggretor agent to generates a comprehensive explanation. There is a significant improvement compared to a single LLM, but the real-time prediction is insufficient. Redman et al. (14) developed a data-driven fetal distress alarm system called OxSys. Afridi et al. (15), Jebadurai et al. (16), and Kadhim et al. (17) focused their research on feature selection. The experimental results showed that after feature selection, the noise in the prediction could be reduced. Alotaiby (18) used Common Spatial Pattern (CSP) to extract features from the original fetal heart monitoring signals for classification. Al-Nussairi et al. (19) shows the application of multi-stage deep learning framework in medical diagnosis.

Ensemble learning is an effective strategy to improve the performance of deep learning model. Mahanty et al. (20) proposed a method combining enhanced xception and snapshot ensemble for Alzheimer's disease detection. Berrones-Reyes et al. (21) proposed a breast cancer detection method combining deep convolutional neural network and fuzzy ensemble modeling technology. Mahanty et al. (22) combined with fuzzy ensemble and transfer learning model for COVID-19 CT image detection. Different integration strategies have a significant impact on the performance of the model. Low et al. (23) compared the effects of majority voting and choquet fuzzy integral in COVID-19 detection.

In summary, the accuracy of traditional machine learning methods are all above 90%. XGBoost often relies on the optimization of feature engineering in tabular data classification tasks, while the MLP algorithm without feature engineering often performs worse than XGBoost. Generally speaking, simple machine learning methods such as Naive Bayes and Random Forest are interpretable, while deep learning methods are often not. In the clinical medical field where patient safety is the core criterion, classification algorithms need to have both high-precision predictive performance and the interpretability.

In this study, an efficient MLP ensemble method called TabM was combined with the PLE algorithm. TabM is used to integrate multiple MLP weak classifiers to achieve the effect of improving the overall performance of the model. While PLE is used to capture the position information of CTG features in different segmented intervals. After comparing with advanced models such as RF, MLP, and XGBoost, this paper introduced the Gradient SHAP analysis technique to explain the model's prediction behavior. Our main contribution consists of three parts: (1) Proving that the PLE algorithm can capture the segment position information of CTG features. (2) After integration, the MLP can achieve performance better than traditional machine learning methods. (3) Using Gradient SHAP to explain the model's prediction behavior reveals consistency with clinical diagnostic standard.

The remain of this paper is organized as follows: The Section 2 is used to analyze the features in the dataset. The Section 3 introduces the techniques employed. Comparisons with other machine learning methods and ablation experiments are presented in Section 4. The analysis of the experimental results is conducted in Section 5. Finally, the Section 7 summarizes the entire paper.

## 2 Dataset description

### 2.1 Feature description

The dataset used in this paper is from the University of California (24), The dataset contains 2,126 samples of fetal heart rate monitoring data. It used the SisPorto 2.0 program (25) to process the original CTG signals and convert them into tabular feature data. These records were classified by three experts according to their postnatal distribution. This dataset has 21 columns of feature data and one column of label data, the meaning of each column is shown in Table 1.

**Table 1:** CTG dataset feature description.

Feature	Description	Min	Max	Mean	Std
LB	FHR (beats per minute)	106	160	133.30	9.84
AC	Accelerations per second	0.00	0.02	0.00	0.00
FM	Fetal movements per second	0.00	0.48	0.01	0.05
UC	Uterine contractions per second	0.00	0.01	0.00	0.00
DL	Light decelerations per second	0.00	0.01	0.00	0.00
DS	Severe decelerations per second	0.00	0.00	0.00	0.00
DP	Prolonged decelerations per second	0.00	0.01	0.00	0.00
ASTV	Percentage of time with abnormal short term variability	12	87	47.01	17.21
MSTV	Mean value of short term variability	0	7	1.34	0.89
ALTV	Percentage of time with abnormal long term variability	0	91	9.88	18.48
MLTV	Mean value of long term variability	0	50	8.21	5.70
Width	Width of the histogram	3	180	70.45	38.96
Min	Minimum of the histogram	50	159	93.58	29.56
Max	Maximum of the histogram	122	238	164.03	17.94
Nmax	Histogram peaks	0	18	4.07	2.95
Nzeros	Histogram zeros	0	10	0.32	0.71
Mode	Mode of the histogram	60	187	137.45	16.38
Mean	Mean value of the histogram	73	182	134.61	15.59
Median	Median value of the histogram	77	186	138.09	14.47
Variance	Variance value of the histogram	0	269	18.81	28.98
Tendency	Histogram tendency	–1	1	0.32	0.61
NSP	Fetal state (0: Normal, 1: Suspect, 2: Pathological)	0	2	–	–

**Figure 1:**
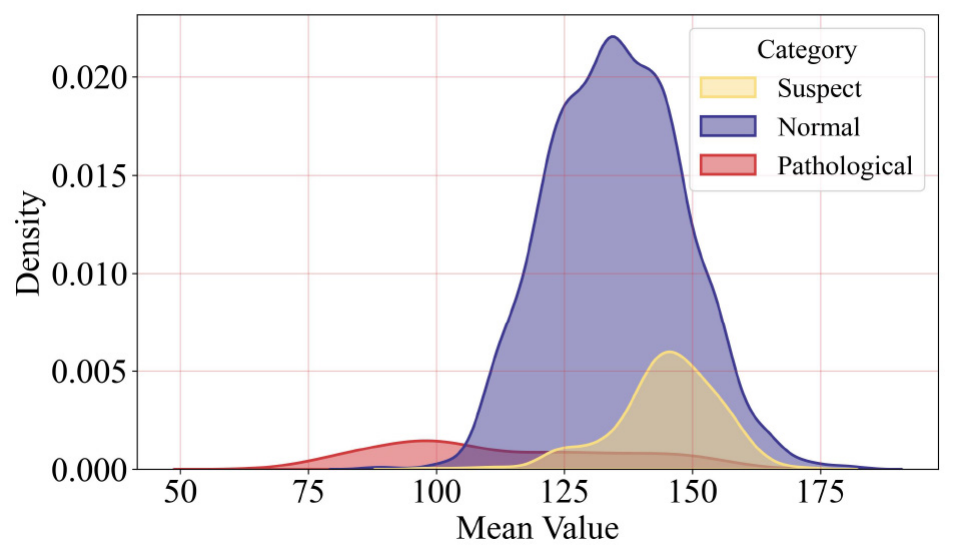
This figure shows the mean feature Kernel Density Estimation (KDE), which indicates that the mean value can effectively distinguish between categories.

Figure 1 shows the distribution characteristics of the mean fetal heart rate in three categories. The distribution range of the normal samples represented by blue is the widest, with the mean value covering the 80–180 interval. And the highest density peak appears at 130–140, indicating that the mean values of most normal samples are concentrated in this interval. The distribution of the pathological samples represented by red is significantly shifted to the left. They only cover the mean value interval of 60–110, with the density peak appearing at 90–100. This distribution has only a small overlap with the normal samples. That phenomenon indicates that a low mean value is a typical feature of pathological samples. So mean value has a good discriminatory ability in distinguishing pathological from noraml samples.

**Baseline**: it refers to the average fetal heart rate within any 10-min period where the fluctuation range is within 5 beats per minute. The baseline reflects the basic heart rate state of the fetus and is regulated by the autonomic nervous system.

**Accelerations**: it refers to a sudden and significant increase in the baseline of the fetal heart rate, with the time from the start to the peak being less than 30 seconds. Acceleration is usually caused by fetal movement or uterine contractions and is an indication of good fetal reserve in the uterus.

**Fetal movements**: fetal movement refers to the physical activities of the fetal in the uterus, such as limb movement and trunk twisting. If the fetal movement decreases or disappears, it is necessary to be alert to the possibility of fetal hypoxia in the uterus.

**Uterine contractions**: the number of uterine smooth muscle contractions monitored during fetal heart rate monitoring is an important indicator for evaluating the progress of labor. Excessive uterine contractions may have potential effects on the fetal, while too few contractions indicate a stalled labor process.

**Decelerations**: temporary drops in the fetal heart rate baseline, classified into three types: mild decelerations, severe decelerations, and prolonged decelerations.

**Short-term variability**: refers to minute fluctuations in the fetal heart rate baseline over several seconds, characterized by rapid, irregular oscillations above and below the baseline. Absence of short term variability may indicate severe fetal hypoxia or neurological suppression.

**Long-term variability**: slow, large periodic fluctuations of the fetal heart rate around the baseline over an extended period.

### 2.2 Data preprocessing

The dataset is divided into training set, validation set, and test set. The training set accounts for 80% (1,700 samples), the validation set accounts for 10% (213 samples), and the test set accounts for 10% (213 samples). Validation set is used for early stop and parameter adjustment. The test set is used only during the test phase. Our early stop strategy is to stop training immediately when the performance of the validation set does not improve for 10 epochs.

The proportion of normal samples is 77.8%, while the rest are suspicious and pathological samples. The fundamental reason lies in real-world medical scenarios where normal infants constitute the vast majority, while abnormal infants represent low-probability events. This data distribution causes the trained model to favor the more abundant normal samples, losing generalization capability for sparsely labeled samples.

**Figure 2:**
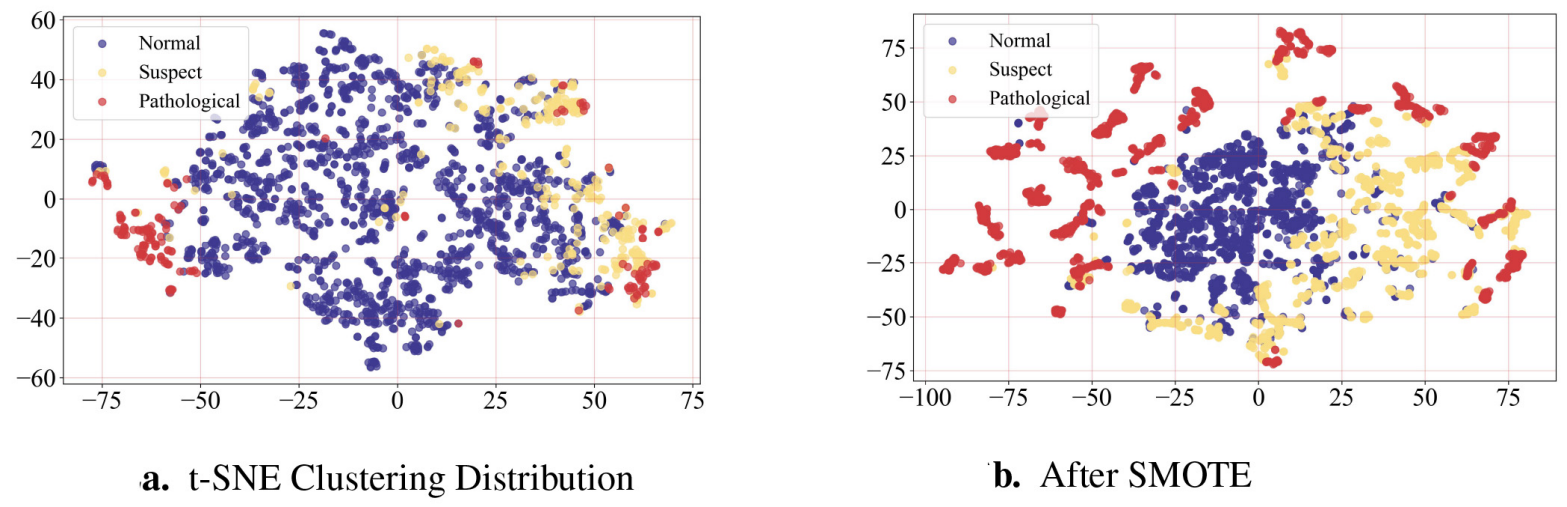
**(a)** Illustrates the clustering distribution of the original data after dimensionality reduction using the t-SNE algorithm. **(b)** Illustrates the clustering distribution of the oversampled data after dimensionality reduction using the t-SNE algorithm.

To address the sample imbalance issue and enable the model to learn sufficiently from sparse samples, this paper used the SMOTE oversampling algorithm to process suspect and pathological samples in the training set. Compared with KMeans, ADASYN and other improved methods, SOMTE's simple algorithm is more suitable for CTG data prediction. SMOTE is an oversampling technique that operates by calculating the *k* nearest neighbors for each minority sample and randomly selecting one from them. The nearest neighbors are computed based on the Euclidean distance between samples. Let the suspect and pathological sample to be synthesized be *x*_*i*_, the nearest neighbor sample be *x*_*j*_, and α be a random number between [0, 1]. The synthesized new sample *x*_*new*_ was generated using the formula shown in Equation 1. By repeatedly applying this process, a large number of samples near the sparse region can be synthesized. Compared to randomly replicating original samples, this approach preserves the spatial feature distribution while expanding the dataset. Applying this method improved model accuracy on suspect and pathological samples. The distribution of the training set data expanded by the SMOTE oversampling algorithm is shown in Figure 2b, where the number of samples in each of the three categories is consistent at 1,326. The t-SNE distribution before dataset expansion is shown in Figure 2a, revealing a small number of suspect and pathological samples. The t-SNE distribution after dataset expansion is shown in Figure 2b, where the number of pathological samples and suspect samples is consistent with normal samples.


$$\begin{array}{l} x_{new}=x_{i}+\alpha *\left(x_{j}-x_{i}\right) \end{array}$$ (1)

The pipeline of data processing is to segment the training set, validation set and test set first. Then standardize on each set. Finally, SMOTE oversampling is performed on the training set.

## 3 Methods

### 3.1 MLP efficient ensemble

The TabM (26) network employed in this paper is based on the MLP architecture. By sharing partial weights to achieve efficient training in the learning tasks, the performance of the model and the training speed can be improved. A single MLP easily overfit on the training set and loses generalization ability on the test set. Training multiple MLPs directly with different random number seeds, and then voting for the predictions can enhance the generalization ability of the model. However, this method results in a large number of parameters for multiple MLPs. Compared to a single MLP, the training time increases linearly. This simple ensemble method also has a drawback: after hyperparameter tuning for a single MLP, the overall ensemble model is not necessarily optimal. In this paper, the method used improves training efficiency by sharing the main weights of the hidden layer within the model. Additionally, adapters were introduced to increase the diversity of sub-models, thereby enhancing the overall performance of the ensemble model.

**Figure 3:**
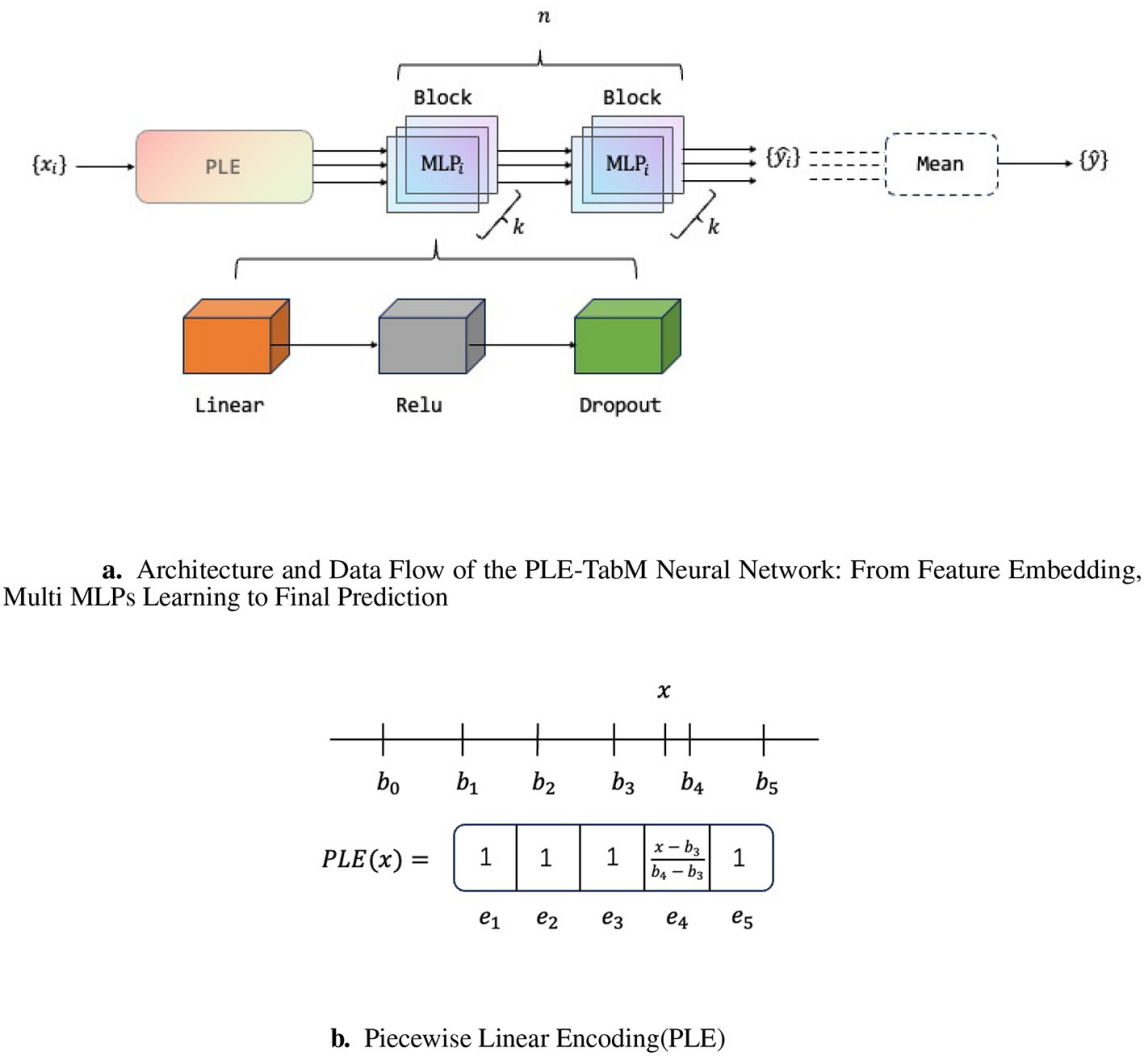
**(a)** Illustrates the overall structure of PLE-TabM model, with *k* copies of each batch data as input. **(b)** shows how to encode a single feature in five bins.


As shown in Figure 3a, TabM is composed of multiple blocks. Each block contains a linear layer, an activation function, and a Dropout layer. Among them, the activation function enhances the model's fitting ability for nonlinear data. The Dropout layer improves the model's generalization ability. The computation for the *i*^*th*^ block is expressed as in Equation 2. The TabM model connects multiple blocks to enhance the model's fitting ability in multiple dimensions. The calculation method of the connection is shown in Equation 3.


$$\begin{array}{l} Block_{i}\left(x\right)=Dropout\left(Relu\left(Linear\left(x\right)\right)\right) \end{array}$$ (2)

$$\begin{array}{l} TabM\left(x\right)=Block_{n}\left(...Block_{1}\left(x\right)\right) \end{array}$$ (3)

In the ensemble phase, *k* MLP layers were integrated within a single block, where *i* denotes the *i*th MLP layer in the block. Any linear layer is denoted as *Linear*(*x*) = *Wx*+*b*, where *W* represents the weights and *b* represents the bias. In conventional deep integration, *Linear*_*i*_(*x*_*i*_) = *W*_*i*_*x*_*i*_+*b*_*i*_, where the *i*th MLP possesses its own weights *W*_*i*_ and *b*_*i*_. In TabM, each MLP shares the common weight matrix *W*. The private weights for the *i*th MLP are constructed using *r*_*i*_, where ⊙ denotes element-wise multiplication. The specific computation process is shown in Equation 4.

$$\begin{array}{l} Linear_{i}\left(x_{i}\right)=W\left(r_{i}⊙x_{i}\right)+b_{i} \end{array}$$ (4)

In the training phase, the training features require *k* copies. Suppose each batch reads m data, with feature dimensions of *d*. The original shape of each batch is (*m, d*). After copying, the data shape becomes (*m, k, d*). At the output layer, the TabM model outputs a feature matrix with dimensions (*k, n*). Here, *n* represents the number of categories. When computing the loss function, TabM calculates the mean loss of *k* weak classifiers using the cross-entropy function. Then optimizes the ensemble model's weights through backpropagation.

### 3.2 Feature embedding

In traditional tabular tasks, numerical features are typically fed directly into neural networks. This paper introduces PLE between the input layer and the TabM to encode features across different intervals, thereby capturing positional information of numerical values within distinct ranges. For a given feature column *x* with data range [*b*_0_, *b*_*T*_], this range can be divided into a total of *T* bins. The original feature *x* can be embedded based on its interval, as shown in Equation 5. Each bin has a learnable feature vector *v*_*t*_, where the position information *e*_*t*_ serves as the weight and the bias is denoted as *b*_0_. As shown in Equation 6, the new encoding *PLE*(*x*) can be computed after linear learning of the numerical feature and embedding of the position feature. When the number of bins degenerates to 1, the PLE embedding layer degenerates into linear feature embedding. A single feature is embedded in the case of five sub bins, as shown in Figure 3b.


(5) $$\begin{array}{lll} PE\left(x\right) & = & \left[e_{1},e_{2},\cdots ,e_{T}\right],\\ e_{t} & = & \{\begin{array}{ll} 0, & x<b_{t-1}\text{and }t>1\\ 1, & x\geq b_{t}\text{and }t>1\\ \frac{x-b_{t-1}}{b_{t}-b_{t-1}} & \text{otherwise} \end{array} \end{array}$$

$$\begin{array}{l} PLE\left(x\right)=Linear\left(PE\left(x\right)\right)=b_{0}+\overset{T}{\underset{i=1}{\sum }}e_{t}\cdot v_{t} \end{array}$$ (6)

### 3.3 Gradient SHAP explanation method

Gradient SHAP is a model interpretation method based on gradients and input feature integrals. Its core idea is to determine feature importance by integrating the model gradient path between input samples and background data (27, 28). In this study, Gradient SHAP was employed to interpret predictions made by the PLE-TabM classifier. Let the proposed PLE-TabM network model be denoted as *F*, where *x*∈*R*^*n*^ represents the current input and *x*′∈*R*^*n*^ denotes background data. The background data in this paper is the training set. For the input feature vector *x* and background data *x*′, the combined gradient for the *i*^*th*^ feature can be expressed by Equation 7. Here $\frac{\partial F}{\partial x_{i}}$ represents the gradient of *F*(*x*) along the *i*^*th*^ dimension.


$$\begin{array}{l} {\text{IntegratedGrads}}_{i}\left(x\right)::=\left(x_{i}-x_{i}^{\prime }\right)\times \overset{1}{\underset{\alpha =0}{\int }}\frac{\partial F\left(x^{\prime }+\alpha \times \left(x-x^{\prime }\right)\right)}{\partial x_{i}}d\alpha \end{array}$$ (7)

## 4 Experimental results

**Figure 4:**
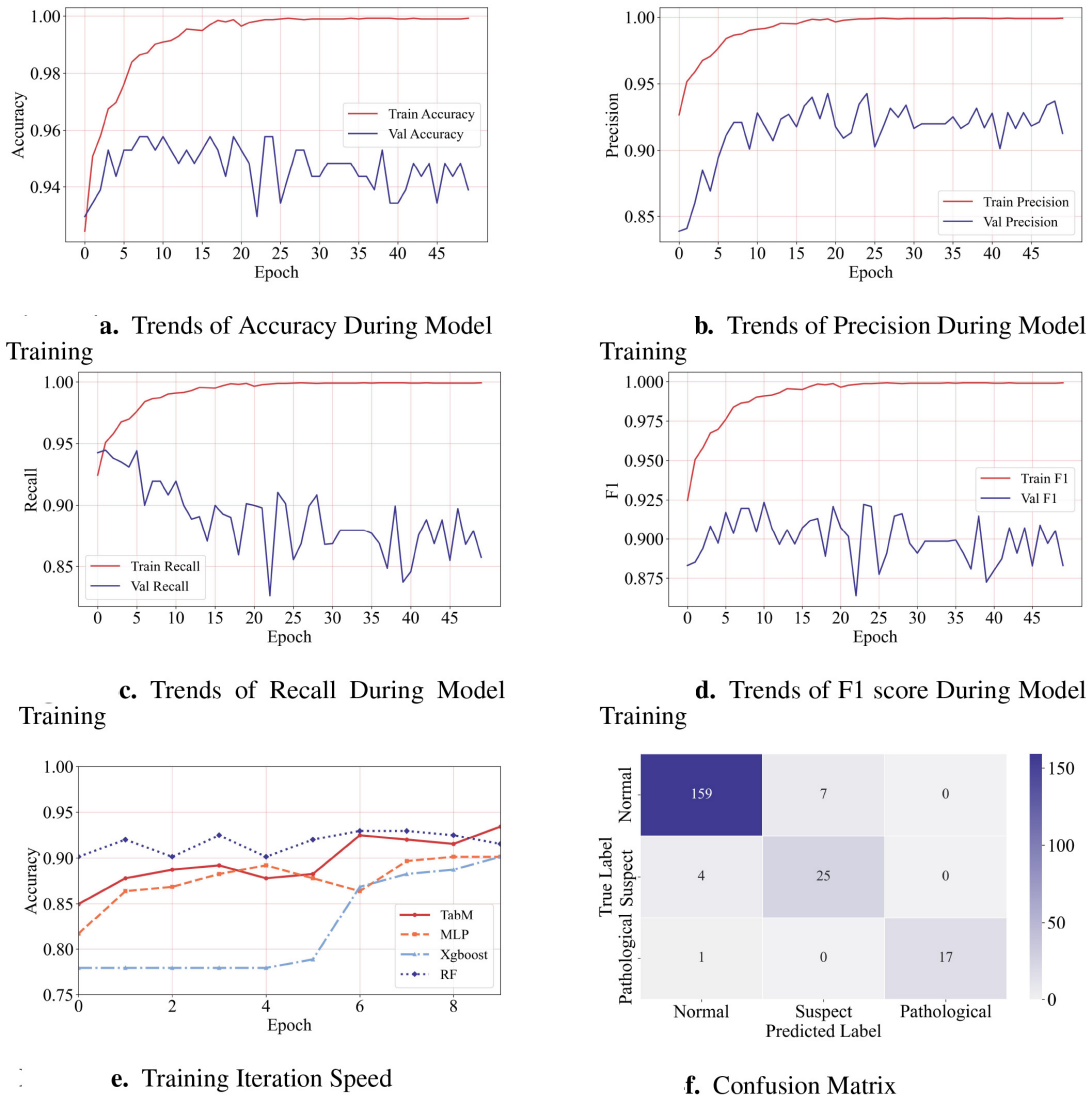
**(a–d)** respectively illustrate the accuracy, precision, recall and F1 score on the validation dataset during the training process. **(e)** illustrates the comparison of the training speed with the baseline model. **(f)** Shows the confusion matrix on the dataset set after the training.

The PLE-TabM was trained on the fetal CTG dataset for 50 iterations. The specific hyperparameter settings are shown in Table 2. The output layer uses Softmax to convert the three dimensions vector of the output layer into a three categories. The loss function uses cross-entropy. All experiments were run on a desktop computer with an AMD R5 5500 CPU and an RTX 5080 16G GPU. During the training process, the evaluation metrics were evaluated on the test dataset. The change process of the evaluation metrics is shown in Figure 4. It can be seen that after 15 iterations of training, the model tends to converge.

**Table 2:** Hyperparameter settings.

Parameters	Values
Optimizer	Adam
Weight decay	1 × 10^−4^
Dropout	0.1
Batch size	64
Patience	10
Learning rate	2 × 10^−3^
k	32
m bins	24
n blocks	2
d embedding	8

The confusion matrix visually represents the alignment between predicted results and true labels, providing an intuitive reflection of the model's classification outcomes. After model training completion, the confusion matrix evaluating the model on the test set is shown in Figure 4f. Among these, four suspect samples were predicted as normal samples, and seven normal samples were predicted as suspect samples. It can be seen that normal samples among suspect samples are relatively prone to confusion, meaning the model lacks sufficient discrimination between a small number of suspect samples and normal samples.

### 4.1 Ablation studies

In complex neural networks, different components and hyperparameters work together to enhance the overall performance of the model. This paper conducted an ablation experiment on hyperparameters, with the parameters changed including the depth *n* of the TabM network, the number *k* of MLPs integrated in a single block, the number *t* of PLE embedding partitions, and the dimension *d* of the PLE embedding feature vector. This experiment is helpful for analyzing which hyperparameters have a crucial impact on the model. This paper conducted an ablation experiment on components,to verify the effectiveness of PLE and TabM.

As shown in Table 3, the number of different block in the model has varying impacts on the overall performance of the model. When *n* = 1, the performance of the model is poor. This shows that it can not reveal the complex patterns in the data. When *n* = 2, all the model's metrics reach their optimum. When *n* = 3, the loss value rises to 0.6381, suggesting that too deep struccture may lead to overfitting, which in turn reduces the model's performance.

**Table 3:** Impact of block count on classification performance.

Exp	N blocks	Loss	Accuracy	Precision	Recall	F1 score
1	1	0.636	0.9014	0.8477	0.8964	0.8639
2	2	**0.6011**	**0.9577**	**0.9333**	**0.9464**	**0.9383**
3	3	0.6381	0.9061	0.8405	0.9078	0.8659

The bolded values indicate the highest classification performance (Loss, Accuracy, Precision, Recall, and F1 score).

As shown in Table 4, the integration of different numbers of MLPs in the model has different impacts on the overall performance of the model. When *k* = 1, the model degenerates into a single MLP. The accuracy and F1 score are the worst. This indicates that a single MLP weak classifier has a limited fitting effect on the data. As the *k* value increases from 1 to 32, it can be observed that the accuracy and F1 score show a gradually increasing trend. This suggests that moderately increasing the number of integrated MLPs within the block can enhance the model's adaptability to different feature spaces.

**Table 4:** Impact of the number of integrated MLP layers on classification performance.

Exp	K	Loss	Accuracy	Precision	Recall	F1 score
1	1	0.6808	0.8685	0.8006	0.8753	0.8191
2	8	0.6033	0.9577	0.9333	0.9464	0.9383
3	16	0.6101	0.9484	0.9176	0.9424	0.9272
4	24	0.6143	0.9296	0.8786	0.9344	0.9013
5	32	**0.6011**	**0.9577**	**0.9333**	**0.9464**	**0.9383**

The bolded values indicate the highest classification performance (Loss, Accuracy, Precision, Recall, and F1 score).

As shown in Table 5, The impact of the number of PLE embedding partitions on the overall performance of the model. When *t* = 24, all the model's metrics reach their peak, which ensures that the partitions are fine enough while avoiding the performance decline caused by excessive partitioning. When *t* = 32, the model's performance drops. Too many partitions reduce samples per partition and increase embedding layer parameters, resulting in overfitting.

**Table 5:** Impact of PLE embedded partition count on classification performance.

Exp	T bins	Loss	Accuracy	Precision	Recall	F1 score
1	8	0.6127	0.9484	0.9176	0.9424	0.9272
2	16	0.6043	0.9484	0.9176	0.9424	0.9272
3	24	**0.6011**	**0.9577**	**0.9333**	**0.9464**	**0.9383**
4	32	0.6215	0.939	0.9113	0.9124	0.909

The bolded values indicate the highest classification performance (Loss, Accuracy, Precision, Recall, and F1 score).

As shown in Table 6, the length of the PLE embedding vector affects the overall performance of the model. When *d* = 8, the model's comprehensive performance is optimal, indicating that an eight dimensionas vector is sufficient for feature representation. When the dimension of *d* increases from 8 to 32, the core indicators continue to decline. This suggests that an excessively high feature embedding dimension will increase the model's parameters, making the model more sensitive to data noise.

**Table 6:** Impact of PLE embedded vector length on classification performance.

Exp	D embeddings	Loss	Accuracy	Precision	Recall	F1 score
1	8	**0.6011**	**0.9577**	**0.9333**	**0.9464**	**0.9383**
2	16	0.614	0.9343	0.8758	0.9174	0.8952
3	24	0.6263	0.9296	0.8786	0.9344	0.9013
4	32	0.6164	0.9296	0.8843	0.9084	0.8936

The bolded values indicate the highest classification performance (Loss, Accuracy, Precision, Recall, and F1 score).

In order to verify the method proposed in this paper, we choose three machine learning algorithms: Random Forest (RF), XGBoost and multilayer perceptron (MLP) for comparison. XGBoost and RF are trained using default parameters. MLP uses two hidden layers containing 128 and 64 neurons respectively, with hyperparameters consistent with TabM. As shown in Table 7, compared with the single MLP model, the TabM algorithm used in this study achieved 90.61%. The TabM algorithm combined with PLE feature embedding is called PLE-TabM algorithm. The table shows that the accuracy of PLE-TabM algorithm is improved by 5% and macro averaged F1 score is improved by 9% compared with TabM algorithm. The Tabm algorithm combining smote oversampling algorithm and PLE feature embedding is called PLE-TabM_*smote*_. The experimental results show that compared with PLE-Tabm, the accuracy of PLE-TabM_*smote*_ is improved by 0.5%, and the macro averaged F1 score is improved by 0.7%.

**Table 7:** Comparison with baseline models.

Model	Accuracy	Precision	Recall	F1 score
Random Forest	0.9343	0.9314	0.851	0.8844
XGBoost	0.9484	**0.9381**	0.895	0.914
MLP	0.9155	0.8969	0.8195	0.8542
TabM	0.9061	0.8218	0.8794	0.8465
PLE-TabM	0.9531	0.9294	0.935	0.9312
PLE-TabM_*smote*_	**0.9577**	0.9333	**0.9464**	**0.9383**

The bold values indicate the highest classification performance (Accuracy, Precision, Recall, and F1 score).

Table 8 shows the classification performance and overall indicators of the model in the three categories of normal, suspect and pathological. In all categories, normal and pathological categories are excellent in accuracy, recall and F1. The performance of the suspect category is slightly lower, but still maintains a high level. The overall classification effect is stable and reliable.

**Table 8:** Each category performance metrics.

Label	Accuracy	Precision	Recall	F1 score	Support
Normal	**0.9639**	0.9816	**0.9639**	**0.9726**	166
Suspect	0.931	0.8182	0.931	0.871	29
Pathological	0.9444	**1.0**	0.9444	0.9714	18
Macro average	–	0.9333	0.9464	0.9383	213
Balanced accuracy	0.9464	–	–	–	–

The bold values indicate the highest classification performance among all categories.

### 4.2 Comparison analysis

In terms of the convergence speed of the model, this paper compares three algorithms: RF, XGBoost and MLP. To clearly observe the convergence speed of the model, only the first 10 iterations are compared here. As shown in Figure 4e, the PLE-TabM model proposed in this study outperforms other algorithms within the first 10 iterations. The figure indicates that RF and XGBoost exhibit slightly slower convergence rate compared to the proposed algorithm, while the single MLP model demonstrates a gradual decline in accuracy during the first 10 training iterations.

In terms of the convergence speed of the model, this paper compares RF, XGBoost and MLP algorithms. In order to clearly observe the convergence rate of the model, only the first 10 iterations are compared here. As shown in the Figure 4e, the convergence speed of MLP and XGBoost is slightly slower than that of the proposed algorithm, while the convergence speed of RF model is faster than that of PLE-TabM.

As shown in Table 9, PLE-TabM exhibits significant improvements compared to existing classification models. Compared to MLP, its accuracy has increased by 3.24%. Compared to XGBoost, its accuracy has increased by 2.77%. Compared to Feng et al. (9) which integrates multiple machine learning algorithms, its accuracy has increased by 0.38% and its F1 Score has increased by 1.34%.

**Table 9:** Comparison with related work.

Paper	Year	Method	Evaluation metric
Mushtaq and Veningston (29)	2024	Random Forest	Accuracy 93%
Salini et al. (30)	2024	KNN	Accuracy 90%
Sirisha et al. (31)	2024	XGBoost	Accuracy 93%
Agarwal and Mohan (32)	2019	SVM	Accuracy 92.39%
Agarwal and Mohan (32)	2019	MLP	Accuracy 92.53%
Feng et al. (9)	2023	Ensemble classifier	Accuracy 95.39% F1 Score 92.49%
Our study	This year	PLE-TabM	**Accuracy 95.77% F1 Score 93.83%**

The bold values indicates the performance in comparison with all related work, achieving the highest Accuracy and F1 Score.

### 4.3 Based on gradient SHAP analysis

**Figure 5:**
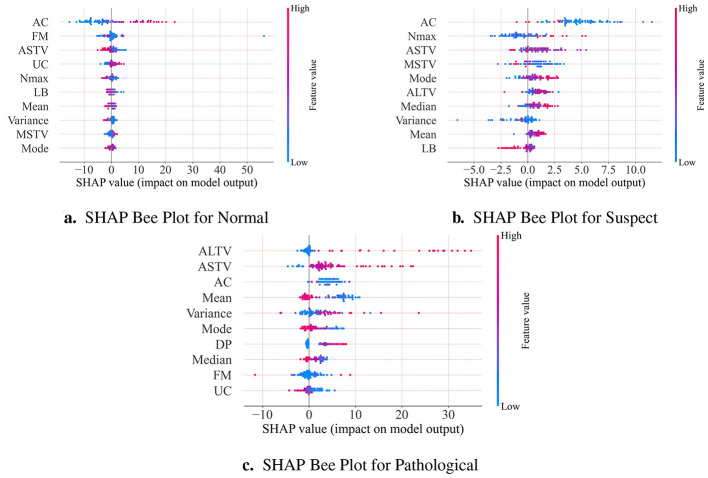
**(a–c)** show the top 10 important features in the three categories of normal, suspect and pathological. The higher the feature position, the greater the impact of the feature on the prediction results.

**Figure 6:**
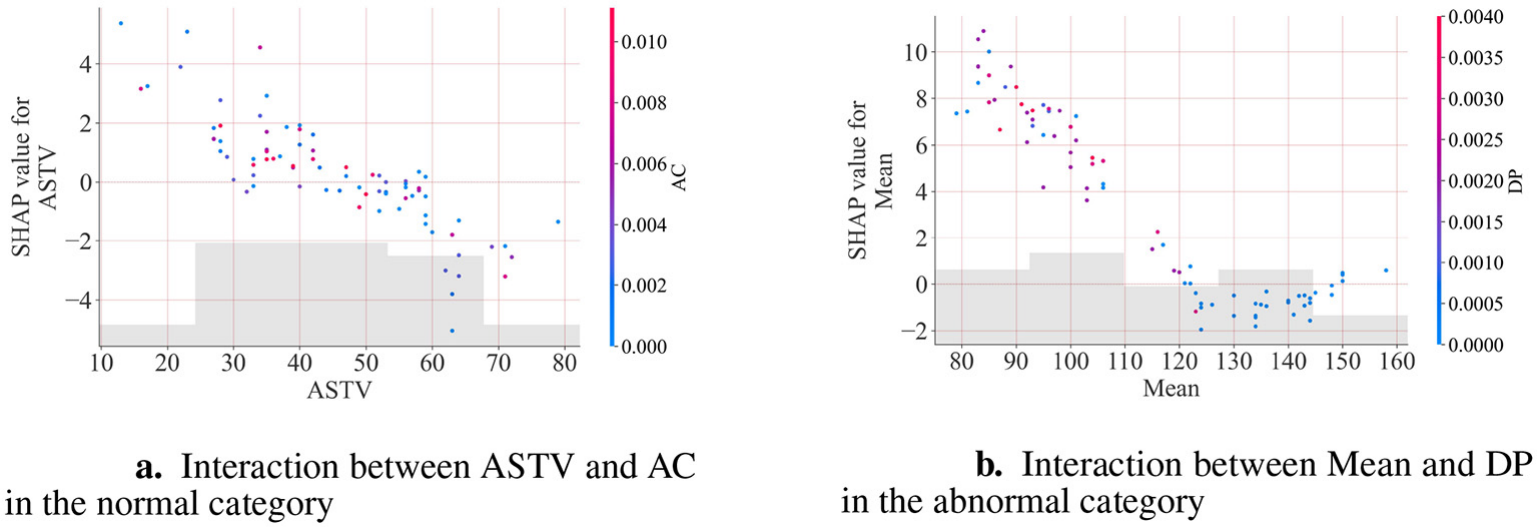
**(a)** and **(b)** illustrate that the two features have a common impact on the prediction.

Figure 5 presents three SHAP value feature importance plots for different fetal heart monitoring classifications. These plots illustrate the contribution of each fetal heart monitoring indicator to the model's classification results through SHAP values. Each point represents the SHAP value of a single feature within an individual CTG sample. The horizontal axis represents the magnitude of a feature's impact on the model output: values closer to 0 indicate less deviation from the reference distribution, while extreme values signify a significant influence of that specific feature. The left vertical axis displays core CTG indicators such as UC, AC, and ASTV, while the color gradient represents the magnitude of the original feature values. Red indicates higher feature values while blue represents lower feature values. This set of graphs provides valuable insights into the behavior of the model, helping to identify the most influential features in each fetal state category. From Figure 6 can be observed that AC, ASTV, UC, MSTV, and FM have the most significant contribution in differentiating between normal and pathological categories; in addition, MEAN, ALTV, AC, ASTV, and DP have the greatest impact on pathological fetal heart monitoring.

### 4.4 Preliminary clinical feasibility assessment

To assess the real world applicability of PLE-TabM, we conducted a preliminary feasibility study using 50 retrospective CTG recordings collected from Hangzhou Red Cross Hospital between January 2025 and June 2025. As shown in Table 10, the reference criteria were developed by three experienced obstetricians with over 10 years of experience, who independently reviewed each CTG according to the FIGO 2015 guidelines. PLE-TabM predictions agreed with the expert consensus in 45 out of 50 cases, achieving an accuracy rate of 95%. This accuracy indicates that PLE-TabM has certain clinical application value. This level of agreement demonstrates promising clinical feasibility, though the small sample size restricts the generalizability of these results to broader clinical populations. These results therefore represent preliminary feasibility evidence rather than robust clinical validation. A prospective and multi center study with a larger sample size is needed to confirm the clinical validity and generalizability of PLE-TabM.

**Table 10:** Preliminary clinical feasibility assessment of PLE-TabM.

Item	Details
Data source	50 retrospective CTG recordings from Hangzhou Red Cross Hospital (Jan 2025 - Jun 2025)
Inclusion criteria	1. Singleton pregnancies with 20 minutes complete CTG recordings
	2. Gestational age greater than 37 weeks
	3. Available definitive postnatal outcomes
Class distribution	Normal: 35 cases (70%)
	Suspect: 10 cases (20%)
	Pathological: 5 cases (10%)
Ground truth	Defined by majority consensus of 3 more than 10 years experience senior obstetricians based on FIGO 2015 guidelines

## 5 Discussion

### 5.1 Model interpretability analysis

Figure 5a shows that AC, FM, ASTV, and UC are core features affecting normal category. These four features exhibit more dispersed SHAP value, indicating more pronounced differences in their contribution. High AC values correlate with elevated SHAP values, significantly increasing the probability of normal category, which is consistent with AC being a direct reflection of fetal intrauterine reserve capacity. In contrast, high ASTV values reduce this probability, as ASTV is a core indicator of fetal nervous system state. FM as an intuitive indicator of fetal vitality, any abnormality in it will directly suggest potential risks to the fetal condition (33).

From Figure 5b, it can be found that in the model prediction results for suspect category, AC, Nmax and ASTV are core distinguishing features, as they are primary evaluation dimensions of fetal status (33). Low AC values correspond to a higher SHAP values, increasing the likelihood of a suspect classification, high values of Nmax and ASTV consistently align with positive SHAP values, suggesting elevated levels of these metrics increase the weight in suspect predictions.

As shown in Figure 5c, for model predictions of pathological category, ALTV, ASTV, AC and Mean are core influencing features, consistent with clinical diagnostic logic of fetal hypoxia and neurological suppression (34, 35). ASTV and ALTV increase under pathological conditions, which may suggest that the fetal nervous system is suppressed or there is intrauterine hypoxia, and thus their SHAP values also increase accordingly (36). Low AC values and low Mean values correlate with high SHAP values have the highest weight in predicting pathological category, as in pathological conditions, AC decreases and fetal heart rate deviations from the normal 110–160 bpm range (37). Furthermore, as can be seen that high DP values, indicating severe decelerations during contractions, also increase abnormal prediction probability, as these decelerations are direct manifestations of fetal hypoxia (38).

### 5.2 Interactions among features

Figure 6a is the SHAP scatter plot of the interaction between ASTV and AC in the normal category: The red dots represent high AC values, which are concentrated in the area where ASTV is lower and the SHAP value is positive; the blue dots represent low AC values, which are concentrated in the area where ASTV is higher and the SHAP value is negative. This indicates that there is an interaction between ASTV and AC: a combination of low ASTV and high AC will enhance the model's prediction of the normal category, while a combination of high ASTV and low AC will weaken this prediction.

Figure 6b is the SHAP scatter plot of the interaction between Mean and DP in the pathological fetal heart rate monitoring category: As the Mean value increases from 60 to 160, the SHAP value changes from positive to negative, indicating that this feature is negatively correlated with the model output. Specifically, a lower Mean (60–110) will have a positive contribution to the prediction, making the model more likely to classify as pathological; a higher Mean (110–160) will have a negative contribution, making the model more likely to classify as normal. Red dots representing high DP values cluster in the region of smaller Mean and positive SHAP values, validating that low Mean values with high DP values correlate with high pathological probability and drive the model toward predicting pathological category.

## 6 Limitation

This paper is only a single dataset study, so the sample is not representative enough. Repeated experiments have not been carried out for many times, and the stability of the model needs to be verified. It is not encapsulated into a runnable desktop program, which is applied to real-time CTG data prediction.

## 7 Conclusion

As machine learning algorithms continue to deepen their application in the medical field, the academic and clinical communities' demands for algorithms have shifted from a simple pursuit of prediction accuracy to exploring the interpretability of black box models. The PLE-TabM algorithm proposed in this paper achieved an accuracy of 95.77% and an F1 score of 93.83%, outperforming traditional machine learning methods. The SHAP interpretation results further confirmed that the PLE-TabM maintains consistency with clinical practice standards in the classification task of fetal cardiotocograph monitoring data. This paper innovatively combines efficient table learning with interpretability analysis and applies it to the CTG problem. It can well meet the core demands of clinical fetal heart rate monitoring for precise judgment and transparent logic, and has high clinical application value in the field of intelligent auxiliary diagnosis of fetal heart rate monitoring.

## Data Availability

The datasets analyzed for this study can be found in the UC Irvin Machine Learning Repository. And the experimental code and data analysis can be obtained from here: https://github.com/connorshen/PLE-TabM-CTG-Research.git.
