# Correction: Fetal health state detection method based on parameters efficient ensembling of deep learning

**DOI:** 10.3389/fpubh.2026.1925419

**Published:** 2026-07-22

**Authors:** Weiwei Yin, Zhengyuan Shen, Zhenbo Cheng, Chun Feng, Guoquan Sun

**Affiliations:** 1 Hangzhou Red Cross Hospital, Hangzhou, Zhejiang, China; 2 College of Computer Science and Technology and College of Software, Zhejiang University of Technology, Hangzhou, Zhejiang, China; 3 The Second Affiliated Hospital, Zhejiang University School of Medicine, Hangzhou, Zhejiang, China

**Keywords:** cardiotocograph, clinical application, deep learning, ensemble learning, piecewise linear encoding

**The Data availability statement** was erroneously given as “The datasets analyzed for this study can be found in the UC Irvin Machine Learning Repository. And the experimental code and data analysis can be obtained from here: https://github.com/connorshen/PLE-344TabM-CTG-Research.git.”

The correct **Data availability statement** is “The datasets analyzed for this study can be found in the UC Irvin Machine Learning Repository. And the experimental code and data analysis can be obtained from here: https://github.com/connorshen/PLE-TabM-CTG-Research.git.”

The original version of this article has been updated.

